# Extracellular Vesicles as Mediators of Cellular Crosstalk Between Immune System and Kidney Graft

**DOI:** 10.3389/fimmu.2020.00074

**Published:** 2020-02-27

**Authors:** Marco Quaglia, Sergio Dellepiane, Gabriele Guglielmetti, Guido Merlotti, Giuseppe Castellano, Vincenzo Cantaluppi

**Affiliations:** ^1^Nephrology and Kidney Transplantation Unit, Department of Translational Medicine, University of Piemonte Orientale (UPO), Novara, Italy; ^2^Center for Autoimmune and Allergic Diseases (CAAD), University of Piemonte Orientale (UPO), Novara, Italy; ^3^Division of Hematology/Medical Oncology, Icahn School of Medicine at Mount Sinai Hospital, The Tisch Cancer Institute, New York, NY, United States; ^4^Nephrology Dialysis and Transplantation Unit, Department of Medical and Surgical Sciences, University of Foggia, Foggia, Italy

**Keywords:** extracellular vesicles, acute rejection, ischemia-reperfusion syndrome, autoimmunity, renal transplant, biomarker, miRNA

## Abstract

Extracellular vesicles (EVs) are known immune-modulators exerting a critical role in kidney transplantation (KT). EV bioactive cargo includes graft antigens, costimulatory/inhibitory molecules, cytokines, growth factors, and functional microRNAs (miRNAs) that may modulate expression of recipient cell genes. As paracrine factors, neutrophil- and macrophage-derived EVs exert immunosuppressive and immune-stimulating effects on dendritic cells, respectively. Dendritic cell-derived EVs mediate alloantigen spreading and modulate antigen presentation to T lymphocytes. At systemic level, EVs exert pleiotropic effects on complement and coagulation. Depending on their biogenesis, they can amplify complement activation or shed complement inhibitors and prevent cell lysis. Likewise, endothelial- and platelet-derived EVs can exert procoagulant/prothrombotic effects and also promote endothelial survival and angiogenesis after ischemic injury. Kidney endothelial- and tubular-derived EVs play a key role in ischemia–reperfusion injury (IRI) and during the healing process; additionally, they can trigger rejection by inducing both alloimmune and autoimmune responses. Endothelial EVs have procoagulant/pro-inflammatory effects and can release sequestered self-antigens, generating a tissue-specific autoimmunity. Renal tubule-derived EVs shuttle pro-fibrotic mediators (TGF-β and miR-21) to interstitial fibroblasts and modulate neutrophil and T-lymphocyte influx. These processes can lead to peritubular capillary rarefaction and interstitial fibrosis–tubular atrophy. Different EVs, including those from mesenchymal stromal cells (MSCs), have been employed as a therapeutic tool in experimental models of rejection and IRI. These particles protect tubular and endothelial cells (by inhibition of apoptosis and inflammation–fibrogenesis or by inducing autophagy) and stimulate tissue regeneration (by triggering angiogenesis, cell proliferation, and migration). Finally, urinary and serum EVs represent potential biomarkers for delayed graft function (DGF) and acute rejection. In conclusion, EVs sustain an intricate crosstalk between graft tissue and innate/adaptive immune systems. EVs play a major role in allorecognition, IRI, autoimmunity, and alloimmunity and are promising as biomarkers and therapeutic tools in KT.

## Introduction

Extracellular vesicles (EVs or microparticles) is a general term that refers to membrane structures released by all cell types through different biogenesis pathways; EVs are secreted after fusion of endosomes with the plasma membrane (exosomes), shed from plasma membrane (microvesicles), or released during apoptosis (apoptotic bodies). These three entities differ in size (exosomes, 30–150 nm; shedding microvesicles, 150 nm−1 μm; apoptotic bodies, 1–5 μm) and partly in content (1–4). In this review, we will employ the umbrella term “EVs” to include all the above-mentioned types of secreted membrane vesicles.

After cellular shedding, EVs are rapidly taken up by neighboring or distant target cells (paracrine and endocrine effects) through a variety of mechanisms, such as endocytosis, phagocytosis/pinocytosis, membrane fusion, and receptor-mediated endocytosis (2).

**Figure 1:**
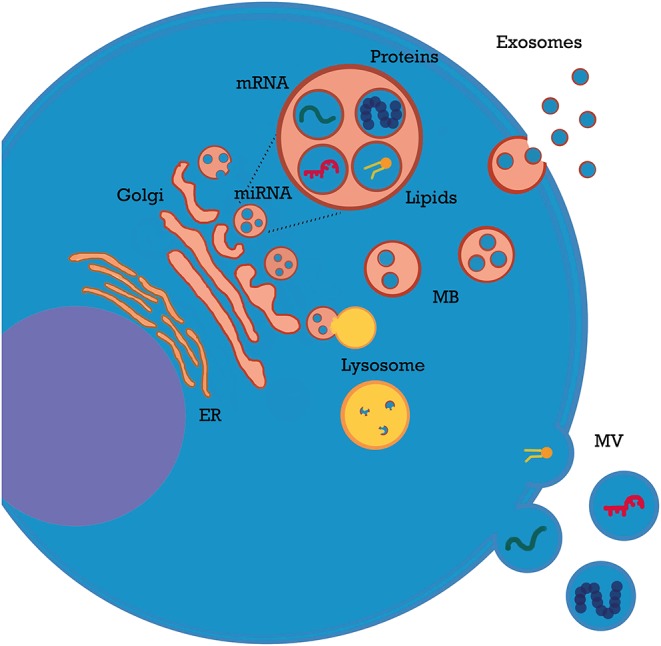
Extracellular vesicle (EV) biogenesis.

EVs are involved in a wide range of physiological and pathological processes (4–7), including acute kidney injury (AKI), chronic kidney disease (CKD), thrombotic microangiopathies, and vasculitis (2, 3). EVs play a key role in all these settings by shuttling their bioactive cargo between cells. Most of their effects are mediated by microRNAs (miRNAs), which modulate gene expression in target cells and induce epigenetic reprogramming (3). Additionally, EVs carry a wide variety of immune modulatory molecules (e.g., cytokines, costimulatory/inhibitory molecules, and growth factors). Packing of nucleic acids and other contents into EVs is coordinated by multiple signals from EVs themselves or from cellular/extracellular environment (8–10). For example, TNFα modulates miRNA content of endothelial particles (11). Of interest, most EVs do not express human leukocyte antigens (HLAs) and escape the immune system; moreover, they cross numerous biological barriers (8), including glomerular endothelium basement membrane (12). Homing and uptake of EVs are mediated by signals and receptors on target cells (13) and influenced by local factors such as pH and electric charge (14). After intake, their complex biocargo exerts multiple effects: mRNAs are translated; miRNAs activate or silence protein expression (1, 2, 8); surface receptors are transferred from one cell to another (15, 16) and bacterial, viral, or graft alloantigens can be exchanged among immune cells (17, 18). A detailed analysis of EV general properties has been covered by recent reviews (1, 6, 8) (Figure 1).

EVs released from innate immune cells, such as macrophages, dendritic cells (DCs), or natural killer (NK) cells, are involved in the regulation of innate immune response mainly as pro-inflammatory and paracrine mediators (4, 19). However, their immunomodulatory role is probably far more complex and includes anti-inflammatory and immunosuppressive effects.

The role of innate immunity as a trigger for acute rejection has been the focus of intense research over the last years (20, 21), and the possibility of manipulating EVs as a therapeutic tool or employing them as biomarkers is opening new paths in solid organ transplantation (22).

The aim of this review is to outline the role of EVs in innate immunity by analyzing different aspects of kidney transplantation (KT) biology. After analyzing EVs as mediators among different innate immune cell types, we will describe the role in complement and coagulation, two pivotal systems in innate immunity, and in other key settings such as allorecognition, ischemia–reperfusion injury (IRI), and the autoimmune component of antibody-mediated rejection. Finally, we will review recent evidence about the role of EVs as potential therapeutic tools and biomarkers in KT.

A general overview of immune-modulating effects of innate cell-derived EVs on different immune system cells or molecular targets is outlined in Table 1.

**Table 1:** Immune-modulating effects of innate cell-derived EVs on other immune system cells or molecular targets.

**Cell of origin**	**Cellular/molecular target**	**EV-surface proteins and content**	**Main biological effects**	**References**
PMN	DC/macrophage	Phosphatidylserine Annexin 1 MPO CD11b/CD18 Lactoferrin Elastase	Reduced DC phagocytosis, maturation and capacity to stimulate T-cell proliferation; reduced production of pro-inflammatory cytokines and increased release of TGF β1 by DC and macrophage (tolerogenic profile)	(23, 24) (25, 26)
	T lymphocytes	CD11b/CD18 Annexin V Elastase	Reduced release of TNFα by naive and activated effector T cells; reduced IL2 secretion and CD25 expression by resting T helper cells	(27) (28) (29) (30) (31) (32)
	Cytokines/membrane receptors	Catepsin G Proteinase 3	Cleavage of cytokines and their membrane receptors	(33) (34)
	L-Arginine	Arginase-1	Reduced T-cell proliferation and function	(30)
	Neutrophil	LT B4 and enzymes for its synthesis; C5R1	PMN chemotaxis	(35) (36)
Macrophage	Macrophage and DC	p-MHC; Microbial and viral antigens; Hsp-70; IL1β; TNFα; CCL2-5; C3 fragments; Proteins of the leukotriene pathway IL 36y miR-223	Transfer of p-MHC, antigens and activating signals to DCs; DC maturation, activation and migration; release of Th1 - (M1 macrophages- derived EVs) or Th2-promoting cytokines (M2 macrophages-derived EVs)	(37) (38) (39) (40) (41) (42)
	PMN	Enzymes of the leukotriene biosynthesis	PMN chemotaxis	(43)
	T lymphocytes	IL1β TNFalfa and CCL2-5 proteins of the leukotriene pathway IL 36y	Increased T cell expansion and differentiation; induction of IFNy and IL 17 producing CD4+ T cells (T helper 17); inhibition of Treg	(40) (44) (45) (46) (35) (47)
	B lymphocytes	C3 fragments IL 36y	Increased B cell expansion and differentiation	(41, 48) (47)
DC	DC	TLR4 p-MHC; Costimulatory or inhibitory molecules; miRNA (miR-148a, miR 451)	Amplification of antigen spreading among APCs and antigen presentation to T lymphocytes	(49, 50) (17, 51) (52)
	PMN	Enzymes of the leukotriene biosynthesis	Neutrophil chemotaxis	(43)
	T lymphocytes	p-MHC; MHC II; Microbial or tumoral antigens; adhesion molecules (ICAM-1); costimulatory molecules (B7 family members)	Activation (mature DCs) or inhibition (immature DCs) of CD4/CD8 pos T lymphocytes	(4, 49) (53) (54) (55) (56)
	B-lymphocytes	Complement fragments, microbial or tumoral antigens		(20, 52)
MC	DC	p-MHC; FcϵRI Hsp 60, Hsp 70; PLA2, PLC, PLD; PGD2,PGE2	Transfer of p-MHC II and IgE-antigens complexes; antigens activation and DC maturation; generation of neolipid antigens	(51) (57) (58) (59) (60) (61)
	T lymphocyte	Proteases	Cytokine inactivation, T helper 2 induction	(51)
	B lymphocyte	CD 40	EVs binding; IL-10 competent B cells	(62)
Eosinophil	DC	MBP EPO	DC maturation; DC-driven Th2 response	(63) (64)
NK	T lymphocyte	Perforin FasL	Cell lysis	(65)

## Neutrophil-Derived Extracellular Vesicles

Far from being mere final effectors of the inflammatory response, neutrophils [or polymorphonuclear cells (PMN)] exert several modulating effects on both innate and adaptive immune cells and can migrate to secondary lymphoid organs. These actions are partly mediated by EVs (23).

In general, PMN-derived EVs have anti-inflammatory and immunosuppressive effects, mainly on DCs and macrophages. EVs released from apoptotic PMNs also extend their actions on T-lymphocyte subsets, blunting their activation (24).

Neutrophil-derived EVs can inhibit lipopolysaccharide (LPS)-activated DCs and macrophages by reducing their phagocytic capacity, their maturation, and the release of pro-inflammatory cytokines (IL-8, IL-10, IL-12, and TNFα) while increasing TGF-β1 excretion. This cytokine plays a key role in suppressing immune response: it promotes anti-inflammatory DC, suppresses CD4^+^ and CD8^+^ T cells and induces T reg expansion (25, 26).

EVs released by apoptotic human PMN suppress T-cell proliferation, IL-2 production, and IL-2 receptor upregulation on activated T cells (27). The binding of these EVs to activated T cells seems to occur through Mac-1 (CD11b/CD18), an integrin also involved in immunological synapse formation (28).

The bioactive cargo of PMN-derived EVs includes numerous immune modulatory molecules: annexin V (induction of Tregs) (29), arginase-1 (depletion of arginine with inhibition of T-cell proliferation), lactoferrin (inhibition of DC migration to lymph nodes) (30), myeloperoxidase (inhibition of DCs) (31), elastase (conversion of human immature DCs into TGF-β1-secreting cells) (32), and other proteases such as cathepsin G and proteinase 3, which can inactivate pro-inflammatory cytokines (IL-2, IL-6, and TNFα) (33) and cleave their receptors from the plasma membrane (34). Additionally, PMN EVs regulate inflammatory cell trafficking; leukotriene B4 (LTB4) activates PMN chemotaxis and is particularly enriched in their EVs (35). Conversely, during sepsis, PNM shed C5a receptor 1 into their EVs and reduce their response to complement activation (36).

## Macrophage-Derived Extracellular Vesicles

In general, macrophage-derived EVs exert pro-inflammatory effects, mainly directed toward DCs, macrophages, PMNs, and T lymphocytes.

Infected macrophages release EVs loaded with pathogens' proteins that can activate other antigen-presenting cells (APCs). Depending on the microenvironment, targeted macrophages activate either M1 or M2 polarization (37), whereas DCs process and present the antigens to T cells, thus promoting allorecognition and adaptive immunity. In addition to microbial or viral antigens, macrophage-derived EVs also carry peptide–major histocompatibility complex (MHC) complexes and costimulatory molecules, further enhancing alloantigen spreading among innate immune cells (38).

The cargo of macrophage-derived EVs includes several molecules with immunomodulatory functions, such as Hsp 70 (pro-inflammatory or tolerogenic effect depending on coexistent signals) (39), IL-1 β (DC migration and expansion of T/B lymphocytes) (40, 41), TNFα, and several chemokines (CCL2, CCL3, CCL4, and CCL5) (44–46). Complement C3 fragments are expressed on EV surface and interact with T cells during antigen presentation (48). Proteins involved in leukotriene synthesis were isolated in human macrophages, converting LTA4 into LTB4 and LTC4 and potentially activating DCs and CD4/CD8 T cells (43). IL-36γ was found in EVs released by infected pulmonary macrophages, with possible impact on DC maturation and T-cell activation [T helper (Th)1 or Th17 development and inhibition of Tregs] (47). Finally miR-223, a regulator of myeloid differentiation, was found in macrophage-derived EVs (42).

Zhang et al. stimulated macrophages *in vitro* with different protocols and performed an extensive proteomic profiling of their EVs. When the inflammasome complex was activated, EVs had a higher immunogenicity and induced NF-κB signaling in neighboring immune cells, thus amplifying inflammation (44). The inflammasome is a multimeric caspase-activating complex that can modulate a wide range of pathways in response to pathogens and activate both innate and adaptive immunity.

This is relevant to KT because IRI determines tissue damage, release of EVs, and inflammasome activation (44). These aspects will discussed in *Extracellular Vesicles in Ischemia–Reperfusion Injury and in the Autoimmune Component of Rejection*.

Finally, glucocorticoid therapy and long-term LPS exposure (mimicking chronic infection) can trigger macrophage release of toll-like receptor-2-containing EVs; these particles act as decoy receptors to antagonize toll-like receptor-2 signaling and blunt inflammation (66).

## Dendritic Cell-Derived Extracellular Vesicles: Early Inflammatory Response and T-Lymphocyte Activation

### Dendritic Cell Extracellular Vesicle and Innate Immunity

DCs highly express pattern recognition receptors and represent a pivotal link between innate and adaptive immunity (49). Toll-like receptors belong to pattern recognition receptors family and play a key role in the early inflammatory response; indeed, toll-like receptors avidly bind damage-associated molecular patterns, a wide group of molecules released by damaged tissues (e.g., during IRI) (67, 68). Toll-like receptor 4 is transferred *via* EVs among bone marrow DCs (BM-DCs) and activate NF-κB signaling pathway (50). Moreover, EV-mediated transfer of miRNAs among DCs contributes to enhance their mutual activation during inflammation (17, 69).

As described above (PMN paragraph), DC-derived EVs also carry enzymes of the leukotriene biosynthesis, which stimulate PMN chemotaxis (43).

### Antigen Presentation to T Lymphocytes

**Figure 2:**
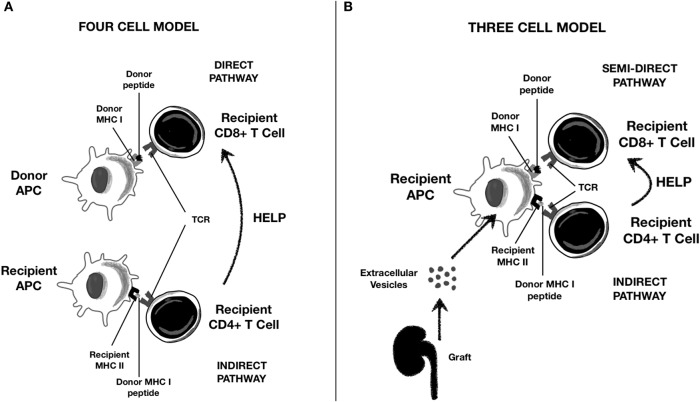
Role of Extracellular Vescicles (EVs) in alloantigen presentation to T lymphocytes. **(A)** Classical direct and indirect presentation; **(B)** “semi-direct” presentation trough cross-dressing of recipient APC with graft-derived EVs.

DC-derived EVs also play a pivotal role in allorecognition (4, 49). DCs capture EVs released from graft tissue. Graft particles carry surface class I and II MHC molecules, non-HLA donor antigens, costimulatory and adhesion molecules, and pro-inflammatory cytokines such as IL-1β (52). The DC–EVs axis plays a pivotal role in all the three antigen presentation pathways described in transplant immunology, as reported in Figure 2 (53, 68, 70, 71):

- Direct antigen presentation: In this setting, donor APCs interact with recipient T cells. Of note, donor DC-derived EVs contain high density of allogeneic peptides complexed with donor MHC (p-MHC) and can interact directly with CD8^+^ and CD4^+^ T cells.
- Indirect antigen presentation: In this pathway, recipient APCs interact with recipient T cells. Graft EVs are internalized into the recipient APC and transfer their peptides to MHC class II molecules. These complexes are then exposed to APC surface for indirect presentation to T lymphocytes.
- Indirect antigen presentation by “cross-dressing” APCs (semi-direct antigen presentation): Donor-derived EVs containing p-MHC complexes are captured by recipient APC on their surface and then presented directly to T cells without any p-MHC reprocessing, a phenomenon referred to as “cross-dressing.”

Recent evidence suggests that donor DC transplanted with the graft are *antigen transporting* rather than *antigen presenting* cells and that “cross-dressing” rather than “passenger leukocyte” is the main mechanism of alloantigen presentation from donor APC (70, 71). Although semi-direct modality rapidly initiates alloresponse and leads to acute rejection, indirect T-cell activation has been associated with chronic antibody-mediated rejection (72). “Cross-dressing” is also typical of follicular DCs, key players in germinal center reactions (54).

The effectiveness of DC-derived EVs in p-MHC presentation depends on the coexistence of other molecules in their cargo (MHC class II, CD86, and ICAM) and on parental cell maturation (20):

- Mature DC-derived EVs are characterized by higher expression of surface MHC, adhesion, and costimulatory molecules (55, 73) and present antigens to CD4^+^ T lymphocytes through “cross-dressing,” promoting Th1 phenotype (56, 74).
- Immature DC-derived EVs are efficiently internalized by mature APCs and transfer their antigens to the target cell MHC. Thus, the antigen is indirectly presented to CD4^+^ T lymphocytes, skewing them toward a Th2 phenotype. Additionally, immature DC can release immunoregulatory EVs loaded with anti-inflammatory cytokines such as TGF-β1 (4) and can target other DCs to amplify a tolerogenic response (75).

Therefore, donor EVs target recipient cells and generate a chimerism that can determine either DC activation or DC inhibition depending on their content (76, 77). For example, EV-derived CD86, a costimulatory molecule, activates T cells through direct or semi-direct pathway, whereas the indirect pathway vehicles miRNAs upregulating PD-L1 and induces CD4 T cells anergy (78). Indeed, graft-infiltrating PD-L1hi cross-dressed DCs blunted T-cell response in a mouse model of liver transplantation (77).

Finally, the relationship between DCs and adaptive immunity is bidirectional: for example, DCs are targeted by Treg-derived EVs that induce a tolerogenic phenotype trough transfer of miRNAs (miR-150-5p and miR-142-3p) (79).

## Mast Cell-Derived Extracellular Vesicles

MC-derived EVs contain p-MHC complexes or endocytosis-derived antigens and can be released by both activated and resting BM-MCs. The main target of these particles is DCs and other professional APCs (51, 80, 81). Skokos et al. investigated the role of MCs in allo-antigen presentation; the authors observed that ovalbumin was more effectively recognized by T cell if taken up by MCs and then transferred to DCs rather than presented directly by DCs (57). Indeed, MCs and DCs form a highly structured immune synapsis devoted to antigen transfer through EVs (58).

Several molecules with immunomodulatory roles have been isolated in MC-derived EVs. Heat shock proteins (Hsp 60 and 70) are essential for antigen loading and EV uptake by DCs (57, 59) and are capable of inducing BM-DC maturation; FceRI–IgE complexes contribute to horizontal antigen transfer among MCs; additionally, phospholipases (PLA-A2, C, and D2), neolipid antigens, and lysophospholipids (60, 61) inhibit DC functions (e.g., phosphatidic acid) and induce Th2 response [lysophosphatidylcholine (LPC)] (60, 82). MC-derived EVs also carry proteases that inactivate cytokines and also target T-cell proteins (34, 60). Finally, CD40L-positive EVs from BM-derived MCs generate IL-10 competent B cells (62, 83).

## Other Innate Immune Cell-Derived Extracellular Vesicles

There is a paucity of data about EVs generated by other innate immune cells.

*Eosinophils* can release EVs containing major basic protein (MBP) and eosinophil peroxidase (EPO) when stimulated with IFNγ; both promote DC maturation (63, 64, 84).

*NK cells* can release EVs loaded with several cytotoxic proteins (85, 86), including perforin and FasL, which can induce lysis of activated T lymphocyte and thus possibly blunt inflammation (65, 87).

The main immune-modulating effects of innate cell-derived EVs on other immune system cells or molecular targets are summarized in Table 1.

## Extracellular Vesicles and the Complement System

EVs can play a dual role in this setting, either activating or inhibiting the complement cascade (88).

This function is extremely relevant to the transplant setting, as EVs play a role in complement attack on ECs in both antibody-mediated rejection (89) and IRI (90).

### a) Extracellular Vesicles as Complement Activators

T cell-derived EVs can activate complement through immunoglobulin binding, whereas other types of EVs do so directly, through interactions between C1q and their membrane lipids (91, 92). For instance, both PMN- and erythrocyte-derived EVs (93, 94) can provide a platform for C1q deposition, with consequent activation of classic pathway on their surface.

Activated endothelial cells can shed EVs under inflammatory conditions; this phenomenon has been observed after complement activation and membrane attack complex formation on endothelial cell surface (95). These endothelial-derived EVs express membrane attack complex and have a strong procoagulant phenotype, which further triggers complement activation through thrombin formation. This creates a vicious circle of endothelial complement-mediated damage and endothelial shedding of complement-enhancing EVs (88).

### b) Extracellular Vesicles as Complement Inhibitors

On the other hand, EV shedding could also represent a mechanism to protect cells from complement attack: indeed, EVs remove complement molecules from cell surface acting as “scavengers” and allowing complement evasion (88). Complement-induced EVs shedding has been demonstrated in PMNs, erythrocytes, and glomerular endothelial and epithelial cells (96).

Consistently, complement-coated EVs from leukocytes can be rapidly phagocytosed by PMNs. Clearance of these opsonized EVs is also facilitated by complement receptor 1, expressed on erythrocytes; as such, red blood cells bind EVs and transport them to the liver and spleen (94).

EVs also carry several complement inhibitors that allow them to transport activated complement factors without being lysed: CR1, CD55, or decay-accelerating factor (modulation of C3 and C5 convertase), CD59 (direct MAC inhibitor), and membrane cofactor protein (MCP or CD46) (2, 97). Interestingly, endothelial EVs are also rich in complement inhibitor mRNA and prevent glomerular injury in experimental models of glomerulonephritis (98).

## Extracellular Vesicles and the Coagulation System

Complement and coagulation cascades are key components of innate immunity and are tightly connected to each other; their simultaneous activation has been extensively studied in transplant rejection and IRI (99–102). EVs released from endothelial cells and platelets (PLTs) are critical promoters of coagulation in renal disease (89); besides carrying inflammatory and chemotactic proteins, these vesicles release also a number of growth factors [e.g., PLT-derived growth factor (PDGF)] and promote tissue regeneration.

### a) Endothelial Extracellular Vesicles

When shed after complement activation, endothelial cell EVs have procoagulant and PLT-activating effects (95, 103). They expose phosphatidylserine and binding sites for factor Va and tissue factor (TF) (104, 105); the latter triggers extrinsic pathway determining thrombin generation (106). Thrombin directly cleaves complement components C3 and C5 into C3/C5 convertase, further amplifying the cascade (107). Endothelial EVs can also transfer TF to monocytes and PLTs (108). On the other hand, these EVs preserve endothelial cell survival in physiological condition (caspase-3 removal and protein C receptor exposure) (109), and EVs derived from endothelial progenitor cells can promote angiogenesis (110).

### b) Platelet-Derived Extracellular Vesicles

These play a key role in hemostasis and coagulation (111) through a variety of mechanisms summarized in Table 2 (112–119). Of note, PLT-derived EVs have a 50- to 100-fold stronger procoagulant/prothrombotic effect than have PLTs (120). On the other hand, they promote angiogenesis and endothelial cell regeneration after vascular injury (2, 121).

**Table 2:** Platelet-derived EVs procoagulant and prothrombotic effects.

**Molecule**	**Mechanism**	**References**
Phosphatidylserine surface expression	Negative charged surface creates binding sites for factors II, Va, Xa (prothrombinase complex)	(112)
Tissue Factor surface expression	It binds factor VIIa on phosphatidylserine- containing surface and activates extrinsic pathway of coagulation	(113)
Protein disulfide isomerase (PDI)	Platelet aggregation	(114)
Receptors for factor VIII	Thrombin generation	(115)
Release of factor XIIa	Activation of intrinsic pathway	(116)
Thromboxane A2 synthesis and release	Platelet aggregation	(117)
IL 1-β release	Monocyte adhesion to endothelium, endothelial cell activation	(118)
RANTES deposition	Monocyte recruitment to endothelium	(119)

## Extracellular Vesicles in Ischemia–Reperfusion Injury and in the Autoimmune Component of Rejection

IRI is the main cause of delayed graft function (DGF), which determines an increased risk of acute rejection and progression to chronic allograft dysfunction (122). IRI triggers a complex, alloantigen-independent immune response characterized by crosstalk between PMNs, macrophages, and DCs (123). All these cells release EVs with pro-inflammatory and anti-inflammatory effects (see above) (19).

Two other cell types release critical EVs in this condition: endothelial cells and renal tubular epithelial cells. Both release EV when exposed to hypoxia, oxidative stress, acidic pH, or inflammation. Hypoxia determines an accumulation of hypoxia-inducible factor (HIF)-α subunit, which dimerizes with HIF-β to form HIF, a transcription factor that can activate over 70 target genes. This results in changes in surface receptors and remodeling of plasma membrane, which triggers release of EVs (124). Furthermore, HIF increases Rab22, an essential element for EV biogenesis (125).

## Endothelial Cells

IRI induces a complex vascular phenotype characterized by a progressive spectrum of functional and structural alterations: vasoconstriction, vascular inflammation, microvascular rarefaction of peritubular capillaries, chronic hypoxia, interstitial fibrosis, and tubular atrophy (126, 127). Microvascular lesions appear to be a key driver of fibrosis after IRI, with a predominant effect over tubular ones (128).

Transplant procedure itself is characterized by tissue damage and some degree of ischemia, resulting in activation of different cell death programs (apoptosis, necrosis, necroptosis, pyroptosis, and autophagy-associated cell death) with release of damage-associated molecular patterns. Bacterial and viral components can also be released during transplant surgery or in infections after KT (122, 123). Both damage-associated molecular patterns and pathogen-associated molecular patterns bind a wide range of innate pattern recognition receptors expressed on several cells, including macrophages, DCs, and endothelia (129). Pattern recognition receptor activation triggers inflammatory response and EV release (126).

Caspase-3 is a pivotal regulator of cell apoptosis (128); under physiological conditions, endothelial EVs protect parental cell by removing caspase-3 (130). During IRI, caspase-3 hyper-activation can overtake EV clearance and cause cell death. In this scenario, endothelial cell generate both “classical” apoptotic bodies and smaller exosome-like vesicles; both are overloaded with caspase-3 and can propagate cell death. Additionally, these exosome-like vesicles carry activated 20S proteasome; this complex recruits adaptive immune cells and induces the production of auto-antibodies toward perlecan/LG3, angiotensin-1 receptor, and dsDNA, further aggravating vascular inflammation (46, 127, 131). Reperfusion has also been associated with the occurrence of a broad range of IgM “natural antibodies,” targeting “neo-epitopes” on ischemic tissues and activating complement (123). Thus, EVs shed by an activated or injured endothelium can trigger mechanisms of alloimmunity and autoimmunity.

The role of EVs in the autoimmune component of rejection has been the focus of recent studies. Tissue-specific self-antigens were found in circulating EVs released by apoptotic cells in the lung, heart, islet, and KT recipients while rejection is developing, whereas they were not detected in control grafts (132). For example, EVs from KT recipients with transplant glomerulopathy have an increased expression of fibronectin and type IV collagen than have EVs from stable KT recipients (133).

Innate immune response generates graft tissue damage, which can favor continuous release of sequestered self-antigens through EVs, with secondary activation of self-reactive T lymphocytes and development of a tissue-restricted form of autoimmunity (46, 72).

It must be emphasized that only in an inflammatory environment (e.g., IRI) can adaptive cells determine autoimmunity. Consistently, Sharma et al. showed that anti-cardiac myosin (CM) antibodies trigger graft rejection in syngeneic heart transplantation only when administered at time of surgery, but not 1 week after it (134).

## Renal Tubular Epithelial Cells

In general, whereas EVs from injured cells can promote tubule interstitial inflammation and fibrosis, those derived from cells with regenerative properties can promote cell proliferation and tissue repair. However, this distinction is blurred, as injured renal tubular epithelial cells can also stimulate repair (as detailed below), whereas mesenchymal stromal cell (MSC)- or endothelial progenitor cell-derived EVs can have harmful effects (135).

With this caveat, we will now focus on actions mediated by EVs released by ischemic renal tubular epithelial cells, whereas EV potential to repair tissue damage will be dealt with in a specific paragraph.

Renal proximal tubular epithelial cells are especially prone to ischemic damage because they depend on mitochondrial metabolism for ATP production owing to their modest glycolytic capacity.

Under hypoxic conditions, HIF-1 mediates EV release by renal proximal tubular epithelial cells (136), which modulate severity of kidney injury by targeting neighboring cells (3).

Furthermore, renal proximal tubular epithelial cells express receptors for complement fractions C3a and C5a and toll-like receptors, making them responsive to innate immune activation (137). Damage-associated molecular patterns can be transferred into renal tubular epithelial cells trough EVs (138) and prevent tubular recovery (139, 140). Pathogen-associated molecular patterns, such as LPS, upregulate the expression of DC-SIGN and toll-like receptor 4, stimulating tubular secretion of IL-6 and TNFα (141).

In this early inflammatory phase, tubular EVs containing cytokines, growth factors, and complement fractions can recruit innate immune cells such as PMNs, M_1_ macrophages, and NK cells (135). EVs released by hypoxic renal tubular epithelial cells are characterized by a decreased content of miR-7641-2-3p, a downregulator of chemoattractant CXCL1, resulting in increased PMN influx (142).

Injured hypoxic tubular cells can transfer TGF-β-containing EVs across disrupted basement membrane to interstitial fibroblasts, activating them and mediating progression to CKD (143). Furthermore, TGF-β itself stimulates renal tubular epithelial cells in an autocrine way to secrete EVs enriched for miR-21, which targets recipient tubules enhancing Akt-mTOR proliferative pathway and consequently exacerbating interstitial fibrosis (144); of note, miR-21 is also released by several other types of human cells through toll-like receptor 3 activation (145).

Also, miR-155 worsens tubular damage during IRI, as it promotes tubular pyroptosis by upregulating expression of caspase-1 and downregulating FoxO3a expression together with its downstream protein “apoptosis repressor with caspase recruitment domain” (146, 147).

In addition to hypoxia, also albuminuria triggers release of CCL2-containing EVs, activating interstitial macrophages and promoting tubule interstitial inflammation (148). Proteases and glycosidase on EV surface may contribute to interstitial fibrosis by degrading extracellular matrix (149).

Furthermore, besides tubular-interstitial diffusion, EVs from renal proximal epithelial cells can also move downstream through urinary tract to target distal tubule or collecting duct (3), although with largely unknown effects.

Tubule-derived EVs can mediate anti-inflammatory and pro-angiogenic actions, for example, secreting IL-10, which polarizes macrophages toward an M_2_ phenotype, and galectin-1 and CD73, which promote Treg function (135, 150–152). Tubule-derived EVs can also directly interact with T lymphocytes through T-cell immunoglobulin- and mucin-containing molecules Tim-1 and Tim-4; interestingly, the same receptor Tim-1 (also called KIM-1) is expressed on renal tubular epithelial cells surface and mediates suppression of NF-κB (153).

EVs also mediate a less defined crosstalk between endothelia and renal tubular epithelial cells. On the one hand, tubule-derived EVs transport ApoA1, which inhibits ICAM-1 and P-selectin and alleviates ischemic damage and PMN retention (154); on the other hand, endothelial EVs can pass into urinary space upregulating HIFα/VEGFα signaling in renal tubular epithelial cells (155).

## Extracellular Vesicles as a Therapeutic Tool in Renal Transplantation

Most studies on EVs as a therapeutic tool in renal transplantation have employed MSC-derived EVs (MSC-EVs) and have focused especially on IRI.

MSCs themselves have drawn much interest in transplantation, mainly because of their capacity to stimulate tissue repair after ischemic injury and their immunomodulatory properties (156).

Injected MSCs can inhibit tubular cell apoptosis and interstitial fibrosis while stimulating proliferation of tissue-specific progenitor cells. Although MSCs can engraft in renal tubular and endothelial cells, regenerative actions are primarily mediated by EVs (157, 158).

Additionally, MSC modulate both innate (DCs, monocytes, and NK cells) and adaptive (T and B lymphocytes) immune cells, with predominantly anti-inflammatory and immunosuppressive effects, which may play a role in preventing or counteracting rejection. Also these effects are predominantly mediated by MSC-EVs (159).

Since side effects and practical challenges of MSC therapy have been reported (160), MSC-EVs have been proposed by several studies as a safer, cell-free alternative. Nevertheless, they have shown similar or even potentially additive regenerative and immunomodulatory properties (161).

Recent evidence suggests that innate immune EVs and MCS EVs play opposite roles in immune system regulation: whereas the former can carry and spread alloantigens, stimulating allorecognition and rejection, the latter can exert immunosuppressive and tolerogenic effects. In particular, MSC-EVs inhibit DC maturation and NK function and skew T lymphocytes toward a Treg phenotype. Of note, MSC-EV proteomic analysis has identified 938 proteins, which could be relevant to MSC-EV interaction with immune cells (159, 162).

MSC-EVs and immune cell EVs are phenotypically different, as they reflect profile of surface molecules of respective parental cell; in particular, MSCs are defined by the expression of CD73, D90, and CD105 and lack CD14, CD34, and CD45 markers (163). However, MSC-EV cargo is not merely a reflection of their parental cell, as it is characterized by a peculiar enrichment in mRNA, miRNAs, and proteins involved in key processes, such as cell cycle regulation, cell differentiation, and immune regulation (157).

### a) Ischemia–Reperfusion Injury

#### Mesenchymal Stromal Cell-Derived Extracellular Vesicles

**Figure 3:**
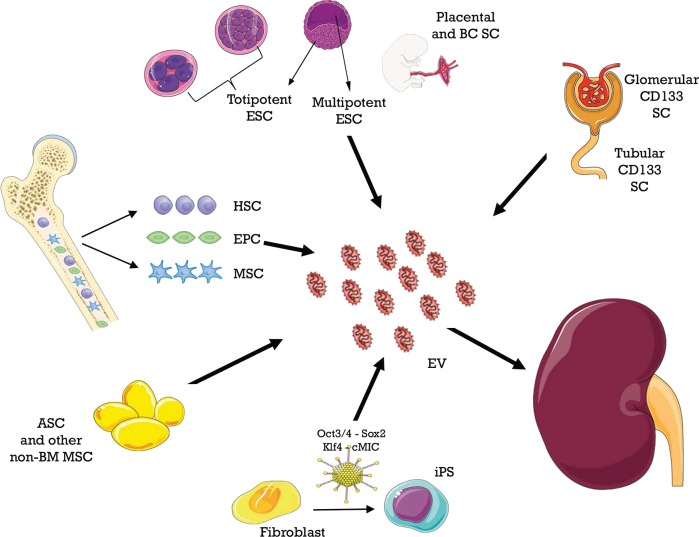
Different sources of extracellular vesicles (EVs) employed as therapy in ischemia–reperfusion injury.

MSCs can be isolated from different tissues, as shown in Figure 3. MSC-EVs recapitulate beneficial properties of origin cells, which are mediated by a variety of mRNAs, miRNAs, and proteins. These molecules are horizontally shuttled into recipient cells and activate signaling pathways related to the following (164):

- renal protection: inhibition of apoptosis/necrosis, inflammation, oxidative stress, fibrogenesis, and promotion of autophagy (165); and
- renal regeneration: stimulation of cell proliferation, migration, tubular dedifferentiation, and angiogenesis.

Importantly, pretreatment with RNAase abolishes these effects, indicating that mRNAs and/or miRNAs account for them (166).

Ferguson et al. identified 23 top miRNAs, which account for over 79% of total miRNA load in MCS exosomes and seem to mediate the predominant effects, targeting 5,481 genes (167). Different miRNAs carried by MSC-EVs are extensively reviewed elsewhere (168–171).

The main miRNAs involved in renal protection from IRI, type of secreting cell, and mechanisms of action are outlined in Table 3 (172–181).

**Table 3:** Main miRNAs involved in renal protection from IRI.

**miRNA**	**Parental cell**	**Mechanism of action**	**References**
miR-125a	Adipose tissue-MSC	Increases endothelial cell angiogenesis	(172)
miR-29b	MSC	Inhibits epithelial mesenchymal transition of rat renal tubule epithelial cells	(173)
miR-21	MSC	Inhibits renal tubule epithelial cells apoptosis and DC maturation	(174)
miR-let7c	MSC	Inhibits renal fibrosis	(175)
mi-R 30	Wharton Jelly-MSC	Inhibits renal tubule epithelial cells mitochondrial fission	(176)
miR-199a-5p	Bone marrow-MSC	Alleviate endoplasmic reticulum stress at reperfusion	(177)
miR-486-5p	Endothelial cell forming colonies	Inhibits endothelial cell apoptosis and endothelial-mesenchymal transition	(178, 179)
miR-218	Renal artery progenitor cell	Increases endothelial cell migration	(180)
miR-126 miR-296	Endothelial progenitor cell	Increases endothelial cell angiogenesis	(181)

Specific functions of these miRNAs are being defined: miR-125a can promote endothelial cell angiogenesis (172); miR-29b inhibits angiotensin II-induced epithelial-to-mesenchymal transition of rat RTECs (173) and blunts inflammation by inhibiting NF-κB; miR-21 prevents renal tubule epithelial cell apoptosis and inhibits DC maturation (174); and miR-199a-5p alleviates endoplasmic reticulum stress at very early reperfusion stages (8–16 h after reperfusion *in vivo*) (177).

Murine studies in which MSC-EVs were employed as a therapeutic tool for IRI are summarized in Table 4 (166, 177, 182–194). In all of them, administration of MSC-EVs improved renal function and/or decreased tubular injury through multiple mechanisms (164). Most studies were performed with BM- and umbilical cord-derived EVs; however, other MSC have been used including kidney resident populations (189, 190) and adipose tissue (191). Of interest, i.v. administered human MSC-EVs were effective in alleviating renal damage in rats that had received KT from cardiac death donor, a procedure characterized by severe IRI (194) (Table 3).

**Table 4:** Studies on MSC-derived EVs as therapeutic tool in AKI from IRI.

**MSC origin**	**Mechanism**	**References**
Human bone marrow	Reduced apoptosis and increased proliferation of renal tubule epithelial cells	(166)
Rat bone marrow	Reduced inflammatory cytokines (IL1β; TNFα)	(182)
Human umbilical cord	Antioxidation through activation of Nrf2/antioxidant response elements (ARE) and decreased expression of NOX2	(183, 184)
Human umbilical cord	Decreased renal fibrosis (downregulation of CX3CL1, decrease of CD68+macrophages); increased angiogenesis (increased expression of renal VEGF)	(185–187)
Human umbilical cord	Tubular cell dedifferentiation and growth (increased ERK1/2 and HGE expression)	(188)
Human umbilical cord	Inhibition of mitochondrial fission (miR-30) and reduced apoptosis	(176)
Mouse kidney resident	Increased proliferation and reduced apoptosis; increased angiogenesis	(189)
Mouse kidney resident (glomeruli)	Increased proliferation of renal tubule epithelial cells	(190)
Rat adipose tissue	Inhibition of oxidative stress, apoptosis, renal fibrosis	(191)
Human umbilical cord	Increased proliferation and fibrosis (releasing from G2/M cell cycle arrest)	(192)
Human bone marrow	Inhibition of apoptosis (downregulation of Sema3A expression and activation of AKT/ERK pathways through miR-199a-3p); inhibition of NK	(193)
Human umbilical cord	Inhibition of apoptosis, increased proliferation of renal tubule epithelial cells; reduced CD68+macrophages infiltration; reduced fibrosis (decreased expression of aSMA and TGFβ; increased expression of HGF)	(194)
Human BM	Suppression of endoplasmic reticulum stress (miR-199a-5p)	(177)

Trophic factors carried in MSC-EVs depend on the parental cell and the surrounding milieu, such as inflammation and hypoxia (136, 164, 195).

Hypoxia has a profound impact on EV properties. In general, ischemic conditioning (preconditioning, postconditioning, and remote conditioning) provides positive results in the setting of myocardial infarction, and hypoxic EVs appear to mediate these effects (124, 125, 195).

Hypoxic EVs derived from BM MSCs can exert protective effects in experimental models of AKI through several mechanisms: inhibition of renal tubule and endothelial cell apoptosis, stimulation of endothelial cell proliferation, reduction of inflammation and PMN infiltration, and inhibition of renal fibrosis (124).

Of interest, hypoxia can stimulate the secretion of EVs by adipose tissue-derived MSCs and can enhance their regenerative properties; specific anti-apoptotic, anti-oxidative, anti-inflammatory and pro-angiogenic pathways are activated by hypoxic EVs, and a distinct proteomic pattern is determined by this type of EVs in renal proximal tubule epithelial cells (196).

In the study by Collino et al. (196), four effects were specifically enhanced in hypoxic EV and could blunt progression of ischemic AKI to CKD: downregulation of fibroblast growth factor receptor 1 (FGFR-1), which mediates TGF-β1-induced epithelial-to-mesenchymal transition, and inhibition of maladaptive repair and fibrogenesis (197); angiogenesis stimulation, alleviating renal microvasculature rarefaction under hypoxia (198); translocation of Nrf-2 into the nucleus, activating antioxidant genes such as HO-1 (199); and downregulation of IL-6, blunting macrophage infiltration and polarization toward a M2 phenotype (200).

Moreover, hypoxic EVs carry respiratory complexes, supporting a non-mitochondrial aerobic metabolism when mitochondrial respiratory capacity is impaired (201); they reestablish intracellular ATP levels and reverse pre-apoptotic changes like histone H2 and H2B upregulation (202); they favor cell proliferation through JNK pathway activation (203) and downregulate calnexin, a NADPH oxidase NOX4-interacting protein, reducing reactive oxygen radical formation (204–206).

However, remote ischemic preconditioning on KT recipients has not proven to be as clinically effective as in ischemic heart disease, and further studies are needed to implement these findings into clinical tools (207, 208).

Another therapeutic approach is MSC transfection with specific miRNA. These engineered EVs proved to be more effective than those derived from naïve MSCs (209).

#### Other Cell Type-Derived Extracellular Vesicles

Cell types other than mesenchymal stromal cells also release reno-protective extracellular vesicles.

Under hypoxic conditions, endothelial colony-forming cells inhibit endothelial cell apoptosis and endothelial mesenchymal transition through EV containing miR-486-5p (178, 179), whereas renal artery progenitor cells increase endothelial cell migration through EV containing miR-218 (180).

Endothelial progenitor cells inhibit capillary rarefaction and progression toward chronic lesions in ischemic AKI; this effect was lost after depletion of pro-angiogenic miR-126 and miR-296 by transfection with specific antagomirs (181).

EVs from renal tubule cells also are capable of accelerating recovery of established renal ischemic damage (210).

### b) Acute Rejection

#### Mesenchymal Stromal Cell-Derived Extracellular Vesicles

Studies using EVs from stem cells and tumors have shown immunosuppressive effects of their transcription factors and miRNAs (159).

In an MHC-mismatched rat model of kidney transplant, injection of recipient MSC-EVs on day 7 after transplant has reduced NK infiltrates and almost completely abolished intra-graft TNFα expression. However, B- and T-lymphocyte infiltrates were higher in EV-treated rats, whereas there was no difference in macrophage populations. Importantly, no difference was observed in antibody response against the donor, which occurred in both groups. These data suggest that MSC-EVs mainly affect some type of innate immunity cells (NK cells and related cytokines, such as TNFα), whereas they do not suppress adaptive immunity and rejection in a strong alloreactive model (162, 211).

#### Immune Cell-Derived Extracellular Vesicles

Immunosuppressive properties of EVs (75–79) could be exploited to inhibit innate component of rejection, for example, skewing DC function and maturation toward a tolerogenic profile (212–214). EVs released from Treg lymphocytes modulated DC maturation and prolonged kidney allograft survival in a rat model (215).

In a study on heart transplant rat model, DC-derived EVs were administered together with LF-15-0195, a DC maturation blocker. This approach determined a donor-specific tolerance with significantly blunted anti-donor proliferative response and chronic rejection, resulting in prolonged graft survival (2, 211).

## Extracellular Vesicles as Biomarkers in Kidney Transplantation

EVs have also been investigated as possible biomarkers in KT. Plasma and urinary EVs have been studied in different transplant settings and will be discussed separately (216).

### a) Acute Rejection

#### Plasma Extracellular Vesicles

Plasma EVs are one of the most promising biomarkers for solid organ transplantation, reducing or even obviating the need for renal biopsy (216–218).

In a recent study, Zhang et al. compared levels of mRNA transcripts carried by plasma EVs of patients with antibody-mediated rejection, T-cell mediated rejection, and no rejection and their related genes, identifying those that were significantly overexpressed in EVs from patients with antibody-mediated rejection. On this basis, they created a gene combination score elaborated from mRNA transcripts of four genes (gp130, SH2D1B, TNFα, and CCL4), which was able to predict imminent antibody-mediated rejection (219).

In a study on 231 KT patients, circulating endothelial microparticles were analyzed before and periodically after KT (up to 2 months); plasma levels increased during antibody-mediated rejection episodes and decreased after therapy, with a slower decline in patients with peritubular capillary C4d staining (220).

In another study, quantification was carried out of plasma C4d^+^CD144^+^ EVs released from endothelial cells associated with antibody-mediated rejection (11-fold increase in concentration compared with that in patients with no rejection), its severity, and response to treatment (over 70% decrease in concentration after successful anti-rejection therapy) (100).

#### Urinary Extracellular Vesicles

In one study (221), 11 proteins were significantly enriched in urinary EVs from patients with T cell-mediated rejection; of note, the association was lost when the whole urinary protein fraction was analyzed. This finding highlighted the impact of “background noise” from uromodulin and proteinuria, suggesting that urinary EVs are a more selective source of biomarkers. Despite this, little evidence has been produced on urinary EV RNAs so far, as most papers have focused on total, cell-derived, or cell-free urinary transcripts (222).

In a more recent study, increased expression of 17 urinary EV proteins was found in patients with T cell-mediated rejection and two proteins—tetraspanin-1 and hemopexin—were proposed as biomarkers (223).

Finally, a urine-based platform termed IKEA (“integrated kidney exosome analysis”), detecting EVs shed by T cells into urine, revealed high levels of CD3-positive EVs in patients with rejection, with an accuracy of over 90% for T cell-mediated rejection (224).

### b) Delayed Graft Function and Other Settings

#### Plasma Extracellular Vesicles

In the already mentioned study by Qamri et al., circulating endothelial microparticles decreased within 2 months of KT, paralleling renal function recovery, only in patients with specific types of causal nephropathies such as diabetic nephropathy or glomerulonephritis secondary to autoimmune disorders (220).

Consistently, Al Massarani et al. found a progressive decrease in serum EV concentration and in their procoagulant activity after KT. This evolution was independent from the type of immunosuppression, whereas it seemed to be influenced by history of cardiovascular disease and CMV infection (225, 226). In a Brazilian cohort of 91 KT patients, PLT and endothelial EV size and concentration were significantly different depending on renal function and time from KT (227).

Taken together, these data suggest that decreased endothelial EVs after KT reflect not only antibody- or T cell-mediated rejection but also improvement of preexisting endothelial dysfunction and of cardiovascular risk factors, paralleling recovery of renal function after KT (227, 228).

#### Urinary Extracellular Vesicles

Urinary EVs have been proposed an enriched source of biomarkers of DGF. For example, neutrophil gelatinase-associated lipocalin expression in EVs was higher than in urinary cells and correlated with DGF (229).

Urinary CD133^+^ EVs appear to be decreased in KT patients with slow graft function and vascular damage, suggesting possible damage to renal stem cell compartment (230).

Likewise, a reduction in urinary aquaporin-1- and aquaporin-2-containing EVs was observed in rat model of IRI, probably reflecting impaired trafficking and expression of these proteins in renal tubule epithelial cells (231), confirming previous finding of decreased abundance of aquaporin-1 in KT recipients in the immediate postoperative days (232).

## Limits, Perspectives, and Conclusions

Despite the large volume of literature, our knowledge of innate immunity EVs is still limited (233). Further studies are needed to widen our understanding in graft antigen spreading and processing by DCs (53, 70–72) and to clarify their tolerogenic potential (75–78). Little evidence has been produced on PMN or macrophage vesicles. Additionally, few studies identified the target genes of EV miRNAs.

Finally, a major limit of EV analysis is the lack of standardization and consistency (234): based on different techniques, diverse markers with almost no overlapping results have been proposed. Of note, most housekeeping genes used for cellular assay normalization (e.g., β-actin or GAPDH) are not consistently expressed in EVs. Normalization of urinary EV proteins with tetraspanins (CD9, CD63, or CD81) is not a validated approach, and mRNA analysis remains problematic (235). Finally, most urinary markers should be standardized for urinary creatinine, but not all studies have adopted this method.

Despite these barriers, EVs appear promising as both biomarkers and therapeutic agents in KT. Enhancement of MSC-EVs therapeutic potential through stimulation with biophysical or biochemical cues (i.e., LPS, hypoxia, inflammatory cytokines, growth factors, hormones such as erythropoietin, nitric oxide, and EVs from other cells such as endothelial cells) is an attractive perspective (236). Genetically engineered EVs overexpressing specific proteins or miRNAs acquire stronger therapeutic properties: for example, HIF-α-overexpressing MSCs have enhanced angiogenic activity and repaired more efficiently cardiac tissue in a mouse model compared with control EVs (237); miR-let7c-overexpressing MSCs selectively homed to damaged kidney, where they upregulated miR-let7c genes and downregulated expression of TGF-β, its receptor (TGF-β-R1), and other pro-fibrotic genes in a renal mouse model of unilateral ureteral obstruction (175).

Bioengineered EVs hold promise as targeted vehicle of drugs or miRNAs, as they naturally overcome biological barriers (209, 238). “Decoy EVs” have also been employed to antagonize inflammatory cytokines (239).

In conclusion, EVs finely tune the crosstalk among innate immune cells and graft tissue; in particular, they determine antigen spreading and “cross-dressing” in the early transplant phases, thus being a key trigger of either alloimmunity or graft tolerance. Systemically, they modulate complement and coagulation cascades during transplant related kidney injuries as antibody-mediated rejection and IRI.

Growing evidence support a potential application of EVs derived from MSCs and other cell types as therapeutic tools in different settings of renal transplantation. Finally, urinary and serum EVs are promising biomarkers of rejection and DGF, opening new paths toward a renal “liquid biopsy.”

## Author Contributions

MQ and VC designed, wrote, and critically revised the Review. SD analyzed innate cells derived EVs. GG and GM analyzed EVs role in DGF and rejection. GC dealt with EVs and complement and coagulation system.

### Conflict of Interest

The authors declare that the research was conducted in the absence of any commercial or financial relationships that could be construed as a potential conflict of interest.
